# Formalising prestige bias: Differences between models with first-order and second-order cues

**DOI:** 10.1017/ehs.2024.12

**Published:** 2024-03-20

**Authors:** Seiya Nakata, Akira Masumi, Genta Toya

**Affiliations:** 1 International Research Center for Neurointelligence (WPI-IRCN), The University of Tokyo, Tokyo, Japan; 2 School of Knowledge Science, Japan Advanced Institute of Science and Technology, Ishikawa, Japan; 3 Research Center for Advanced Science and Technology, The University of Tokyo, Tokyo, Japan

**Keywords:** Computational model, cultural evolution, indirect bias, prestige, social learning

## Abstract

Knowledge and behaviour are transmitted from one individual to another through social learning and eventually disseminated across the population. People often learn useful behaviours socially through selective bias rather than random selection of targets. Prestige bias, or the tendency to selectively imitate prestigious individuals, has been considered an important factor in influencing human behaviour. Although its importance in human society and culture has been recognised, the formulation of prestige bias is less developed than that of other social learning biases. To examine the effects of prestige bias on cultural evolution theoretically, it is imperative to formulate prestige and investigate its basic properties. We reviewed two definitions: one based on first-order cues, such as the demonstrator's appearance and job title, and the other based on second-order cues, such as people's behaviour towards the demonstrator (e.g. people increasingly pay attention to prestigious individuals). This study builds a computational model of prestige bias based on these two definitions and compares the cultural evolutionary dynamics they generate. Our models demonstrate the importance of distinguishing between the two types of formalisation, because they can have different influences on cultural evolution.

## Graphical abstract

**Figure:**
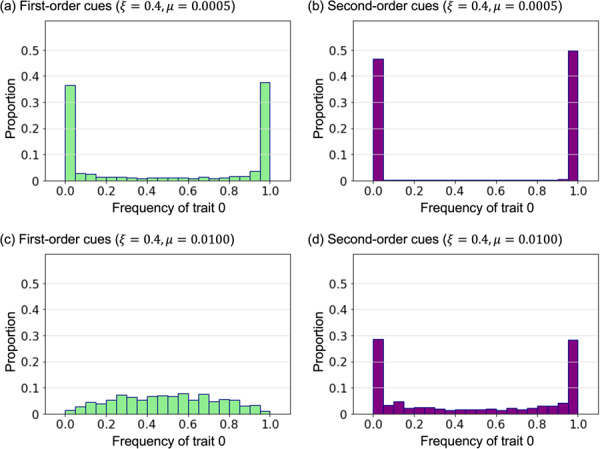

**Social media summary**: Prestige-based social learning is important for human cultural evolution, but needs to be carefully formalised.

## 1. Introduction

It has been argued that selective social learning bias is key to human ecological success (Boyd & Richerson, 1988; Henrich & Boyd, 2002). Various types of social learning biases have been reported in various animals (Hoppitt & Laland, 2013), although prestige bias may be unique to humans. Prestige bias is the tendency to selectively copy cultural traits (e.g. behaviours and beliefs) possessed by individuals who are highly respected and admired. Henrich and Gil-White (2001) developed the theory of prestige from evolutionary views. Many researchers have tested the predictions derived from their theories (cf. Jiménez & Mesoudi, 2019). Anthropologists, biologists and psychologists have empirically tested predictions of which individuals will be considered prestigious and behaviours towards prestigious individuals. This study focuses on prestige-biased social learning and cultural evolution. There is a vast body of literature on the relationship between social learning biases and cultural evolution. Some researchers argue that prestige bias causes remarkable cultural dynamics such as maladaptive cultural evolution (Ihara, 2008; Mesoudi, 2009b) and cumulative cultural evolution (Henrich, 2015). However, there is limited theoretical work on the impact of prestige bias on cultural evolution than on other social learning biases. We reviewed the definition of prestige bias in previous studies and constructed two computational models for prestige-biased social learning. We argue that these two models have led to different patterns of cultural evolution. Formalising prestige bias will allow us to understand some remarkable cultural evolutions in humans.

This study aims to understand the cultural evolution shaped by prestige bias. Section 2 summarises the characteristics of prestige bias by comparing it to other social learning biases. Section 3 reviews the definitions of prestige bias in previous research and describes the two types of prestige bias, while Sections 4 and 5 model these two types of prestige bias and simulate how they differ in cultural dynamics. Finally, Section 6 suggests directions for future research.

## 2. Distinguishing prestige bias from other social learning biases

Many animals use both individual learning by observing or interacting with the environment, and social learning by observing or interacting with other individuals. Individual learning is costly but provides current information regarding the environment. Social learning is a means of acquiring adaptive information at a low cost; however, it may also lead to the acquisition of outdated information (Laland, 2017). Traditionally, researchers have believed that social learning and the culture transmitted through social learning increase human biological fitness. However, Rogers (1988) showed that random social learning does not necessarily lead to greater fitness than individual learning. Based on these findings, researchers focused on selective social learning. The tendency to select who to learn from, when and what to learn is characterised as social learning bias or social learning strategy (Boyd et al., 2011; Boyd & Richerson, 1988; Henrich et al., 2008; Henrich & McElreath, 2003). A variety of social learning biases have been examined (Hoppitt & Laland, 2013; Rendell et al., 2011), including prestige bias. Social learning biases are classified into three types in the cultural evolution literature: trait-based, frequency-dependent and demonstrator-based. Prestige bias is considered as demonstrator-based. In the following sections, we will show that other social biases have made progress in theoretical study, and we will discuss the place of prestige bias in demonstrator-based social learning.

### 2.1. Trait-based bias and frequency-dependent bias

When cultural learning selection depends on the characteristics of a cultural trait, it is labelled as direct bias, content bias, ‘what’ strategy or trait-based bias. Supposing that a social learner observes various types of tennis rackets in a population. If the learner is more likely to choose larger rackets without considering who owns them, then the learner has a trait-based bias. Psychological studies have identified several candidates for trait-based biases in cultural transmission (content that matches gender stereotypes: Bangerter, 2000; Kashima, 2000; structured strings: Kirby et al., 2015; emotional content: Eriksson et al., 2016). Mathematical models suggest that trait-based biases for higher fitness can promote cumulative cultural evolution (Mesoudi, 2011b).

When cultural learning selection depends on the frequency of a cultural trait, it is labelled as frequency-dependent bias. In the cultural evolution literature, one of the most cited social learning biases is conformity bias (also labelled positive frequency-dependent bias or copy-the-majority), which is the tendency to copy the majority of cultural traits. Social psychologists have repeatedly reported that people tend to conform to majority opinion (Mesoudi, 2009a). Rigorously, conformity bias is formulated as the bias to copy disproportionately more of the most frequent cultural traits in a population regardless of the content or possessor of the trait. Conformity bias is often described as an independent category or a subcategory identical to demonstrator bias described in the next section (also labelled indirect bias, context bias or ‘who’ strategies). However, because the selection targets of conformity bias in many studies are cultural traits rather than demonstrators, it can be categorised as trait-based bias. Studies using mathematical models have shown that conformity bias evolves when the population size is large (King & Cowlishaw, 2007), the cost of individual learning is low and the environment is unstable (Nakahashi, 2007). Laboratory experiments have shown that humans exhibit behaviours that follow conformity bias in similar situations where conformity bias theoretically evolves (Kameda & Nakanishi, 2002; McElreath et al., 2005; Toyokawa et al., 2019). Fitting experimental data to computational models has shown that when people can use success and conformity biases simultaneously, they often prefer success bias to conformity bias (McElreath et al., 2008).

### 2.2. Demonstrator-based bias

When cultural learning selection depends on the characteristics of the possessor of a cultural trait, it is labelled indirect, model or demonstrator-based biases. The possessor of a cultural trait observed by social learners is called the model or demonstrator. For example, if a social learner is more likely to choose a racket used by a player from the same hometown, then the social learner has demonstrator-based bias. Empirical studies have identified several candidates for demonstrator-based biases in cultural transmission (familiarity-based: Corriveau & Harris, 2009; kin-based: Henrich & Henrich, 2010).

Many studies on cultural evolution have focused on success and prestige biases among demonstrator-based biases. Success bias is also called payoff bias or ‘copy most successful individual’. An individual following success bias selects the most successful demonstrator and copies its cultural traits. ‘Success’ is defined biologically (e.g. reproductive success) or culturally (e.g. economic success). This bias is adaptive when the environment is complex (Mesoudi, 2008). Success bias homogenises cultural traits within a group (Atkisson et al., 2012; Mesoudi, 2011a; Mesoudi & O'Brien, 2008).

Although success bias contributes to individual fitness, direct information on success is often ambiguous (Hill & Kintigh, 2009). In such cases, the social learner must rely on other characteristics of the demonstrator that may lead to success. Prestige is an indirect cue for success. Selective social learning based on prestige is labelled prestige bias. Individuals are influenced by prestigious people when making decisions. For example, suppose there are two economics textbooks that are not part of your major. The first was written by a famous Nobel Prize-winning economist. The author of another textbook is someone you do not know. In such situations, you are likely to choose the first option. The fact that our decisions are influenced by prestigious individuals (e.g. Nobel Prize winners) has been studied for a long time, mainly in the field of social psychology (Cialdini & Goldstein, 2004). Social psychology has developed a body of knowledge on how and under what circumstances people are susceptible to prestige effects. In recent years, the impact of prestige on the digital environment has also been studied. The proliferation of digital media such as social networking sites may have increased the impact of prestige on people, as evidenced by the widespread use of the term ‘influencer’ (Acerbi, 2016, 2019).

Descriptive theories of prestige bias by Henrich and Gil-White (2001) predict various phenomena. Over the past two decades, researchers have empirically tested the predictions of Henrich and Gil-White's theory of prestige bias (Jiménez & Mesoudi, 2019). Some researchers have constructed mathematical models to study specific issues of prestige bias, such as the diffusion of cooperative behaviour (Henrich et al., 2015) and maladaptive culture (Ihara, 2008; Mesoudi, 2009b). However, definitions of prestige and prestige bias vary widely across studies. To the best of our knowledge, there is no consensus regarding this formulation. Mathematical and computational modelling studies on prestige bias are scarce.

### 2.3. Towards modelling prestige bias

As noted above, researchers have studied social learning bias from various perspectives. The combination of empirical and theoretical approaches has facilitated an understanding of the evolutionary issues of success and conformity biases. Although a wealth of empirical findings have been accumulated on prestige bias since Henrich and Gil-White (2001), basic research using mathematical models is less advanced. Formulating prestige bias based on empirical findings and conducting mathematical studies can facilitate its theoretical understanding. Further basic research on prestige bias is essential for understanding human uniqueness in the animal kingdom. Therefore, in the next section, we review the definitions of prestige bias used in previous studies to provide generalised formulations.

## 3. Two types of definitions of prestige bias

Prestige-biased social learning is important not only in psychology but also in biological and cultural evolution. This is because human culture is the key to human ecological success, and studying social learning biases can help us understand how cultures evolve (Boyd & Richerson, 1988; Henrich & Boyd, 2002; Laland, 2017). The study of prestige-biased social learning from an evolutionary perspective was initiated by Boyd and Richerson (1985), and later developed by Henrich and Gil-White (2001). These hypotheses have been tested in many empirical studies by anthropologists and psychologists (Jimenéz & Mesoudi, 2019 for a review). Each study uses a different operational definition of prestige. However, we can classify all definitions into two types: prestige based on first- or second-order cues.

### 3.1. Prestige bias based on first-order cues

Prestigious individuals have a powerful influence on people. Research in anthropology and psychology has empirically examined individual characteristics associated with being perceived as prestigious. Prestige cues that are attributed to a demonstrator are referred to as first-order cues. For example, a correlation between generosity and prestige has been reported through experiments (Halevy et al., 2012) and ethnographic observations (Garfield et al., 2019). Generosity would be a good indicator of a demonstrator for copying, because it is difficult to generously distribute one's meagre wealth. The successful individual is likely to be generous, which increases their prestige. An individual's confident behaviour (Anderson & Kilduff, 2009; Jiménez & Mesoudi, 2021) and display of pride (Tracy et al., 2013) may also be related to social status. Other studies have suggested that prestige is associated with job titles (Burris, 2004; Jiménez & Mesoudi, 2020), academic titles (Dalmaso et al., 2012) and wearing certain types of clothing (DeWall & Maner, 2008). Although people can access these cues with less cognitive demand than direct evaluations of success, the cues are also more likely to be unrelated to actual competence and less reliable cues (Jiménez & Mesoudi, 2019).

Population genetics approaches often formalise prestige bias as mathematical models based on first-order cues and the findings of empirical studies. In mathematical models, the differences in prestige among individuals are controlled by exogenous parameters. In such models, a limited number of individuals in a population have high prestige owing to their characteristics (competence, wealth, or appearance). Prestigious individuals have greater social influence than others, and their cultural traits are more likely to be copied. Boyd and Richerson (1988) and Henrich and Boyd (2002) pioneered mathematical models of prestige bias. However, their model assumed that the prestige bias is the tendency to copy successful demonstrators. Given that prestige bias is based on the cues that are unclearly related to actual success as described above, their model should be characterised as a ‘copy successful individuals’ strategy (Mesoudi, 2008).

Some studies have formalised prestige bias independent of success. These studies introduced prestige bias into their models to examine specific questions. For example, Ihara (2008) developed a mathematical model to examine the conditions under which prestige-seeking behaviour evolves while sacrificing reproductive success. The analyses suggest that even costly prestige-seeking behaviours evolve if there is a statistical association between social status and prestige-seeking behaviours. Mesoudi (2009b) developed a mathematical model suggesting that copycat suicide may be caused by prestige bias and other several social learning mechanisms. These two studies specifically focused on the negative aspects of the prestige bias that can spread maladaptive cultures. Meanwhile, Henrich et al. (2015) developed a culture–gene coevolutionary model to investigate the conditions under which prestige promotes the evolution of cooperation.

### 3.2. Prestige bias based on second-order cues

People generally emphasise and learn from prestigious individuals (Henrich & Gil-White, 2001). Social learners can use the extent of attention and the amount copied from others as cues for prestige. These are known as second-order cues, defined not by features attributed to the demonstrator, but by the behaviour of other individuals towards the demonstrator. Brand et al. (2020) replicated the emergence of prestige and prestige biases based on second-order cues in their experiments. Several other experimental studies have employed prestige bias based on second-order cues (Atkisson et al., 2012; Brand et al., 2021; Brand & Mesoudi, 2019; Chudek et al., 2012).

Second-order cues may emerge based on the success of the demonstrators (Henrich & Gil-White, 2001). This makes sense, given the existence of success bias. Social learners are more likely to copy successful demonstrators than others. Several experimental studies have suggested that people selectively copy demonstrators following a success bias (Atkisson et al., 2012; Burdett et al., 2016; McElreath et al., 2008; Mesoudi, 2008; Wood et al., 2013). Successful information causes bias in the distribution of copies and attention. Specifically, a second-order prestige cue emerges. A recent experiment conducted by Brand et al. (2020) showed that participants used information on the number of copies, based on success bias, as a cue for further social learning. The experiment showed the emergence of prestige bias through success bias. However, to the best of our knowledge, no mathematical model has been developed for prestige bias based on second-order cues.

### 3.3. Gaps between two types of prestige bias

Prestige bias has been studied using various approaches, including laboratory experiments, field studies and mathematical models. Although there are variations depending on the purpose of the research or practical reasons, the definitions of prestige bias accepted by researchers fall into two categories: first- and second-order cues based. Prestige bias based on first-order cues is defined by demonstrator characteristics. It includes the demonstrator's behavioural and social labels, which seem to make it easier for learners to recognise high or low prestige without spending time on interactions (e.g. behaving generously or holding a title). Prestige-biased social learners’ selection of demonstrators based on first-order cues is theoretically independent of the behaviours of other learners. Prestige bias, based on second-order cues, is defined as the behaviour of other individuals towards a demonstrator. For example, social learners are more likely to select demonstrators who are imitated by the majority. This can be interpreted as conformity bias in the demonstrator selection process. Therefore, a positive feedback loop similar to that seen in conformity bias would occur, and the value of prestige based on second-order cues held by an individual would change over time.

Prestige bias is implemented so that the culture possessed by more prestigious demonstrators is more likely to be copied than that possessed by less prestigious demonstrators. Previous studies have modelled prestige bias based on first-order cues, and the value of an individual's prestige is fixed from birth to death. The usual assumption for models of demonstrator-based bias is that the parameters for influence are constant (e.g. Acerbi et al., 2022). This would help examine the causal effects of bias on cultural dynamics. However, mathematical models focusing on prestige bias based on first-order cues have only been built to study a limited set of questions (e.g. Ihara, 2008; Mesoudi, 2009b). A prestige bias model based on second-order cues should consider prestige value as a dynamic parameter that varies continuously and temporarily. With a prestige bias model based on second-order cues, researchers can examine the dynamics of both the distribution of prestige value and cultural frequency; however, such models have yet to be constructed.

As discussed above, prestige bias based on first- and second-order cues has different properties in terms of model assumptions. Therefore, the two types of prestige bias may generate different cultural dynamics and explain the different ranges of the phenomena. To understand its nature, we formulated two types of prestige bias and examined the cultural dynamics they produce using simple simulations.

## 4. Computational models of prestige bias

In this section, we present two types of computational models for prestige bias: those based on first- and second-order cues. This study investigates the effect of prestige bias on the dynamics of cultural evolution. In addition, it attempts to answer the question as to whether two formulations of prestige, first- and second-order cues, lead to different outcomes, and if so, what the difference would be. Therefore, agent-based simulations were conducted using these models.

We assumed a simple situation where there are only two types of cultural traits, 0 and 1, for the simulations in this study. The cultural traits for each agent at *t* = 0 were determined randomly. The cultural traits for each agent can change to another owing to mutation after prestige biased social learning (described later). We used various values of mutation rate (*μ*) for our simulation.

### 4.1. Prestige bias based on first-order cues

In this section, we introduce a prestige bias model based on first-order cues. Prestige bias is a demonstrator-based type of bias. Therefore, each agent does not select a cultural trait but a demonstrator at time *t*. For cultural traits, each agent imitates the demonstrator's choice of cultural trait after selecting a demonstrator. The probability that each agent selects a demonstrator *k* = {1, 2, …, *N*}, *q*_*k*_, is defined as follows:1
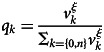


where *v*_*k*_ denotes the prestige value of agent *k*. In this model, *v*_*k*_ is a given value that does not change over time, and probability *q*_*k*_ is constant, where *n* is the number of potential demonstrators from which an agent can select. In this study, we set *n* = *N*; all of the agents are potential demonstrators of someone else, including themselves (when agents choose themselves, it means that they do not change their behaviour). Furthermore, *ξ* is the strength of prestige bias, *ξ* = [0, ∞]. If *ξ* = 0, then each agent can be selected uniformly at random as a demonstrator. If *ξ* > 0, the agents with higher prestige value are more likely to be selected as demonstrators than agents with lower prestige value. After selecting a demonstrator, each agent imitates the demonstrator's choice of cultural traits.

As mentioned above, the prestige of each agent, *v*_*k*_, is given as a constant value over time; thus, we need to specify the prestige value of each agent. For simplicity, we assume that all agents have either a high or a low prestige value. When *n* = *N*, the ratio of high and low prestige values is what matters in this model, not their absolute values. In this study, we set the high prestige value to 10 and the low prestige value to 1, i.e. the ratio is 10. We also determine the proportion of agents with high prestige values. In general, very few individuals in a population have high prestige values (Acerbi et al., 2022). Accordingly, this proportion was set to 10%.

### 4.2. Prestige bias based on second-order cues

Here, we introduce a prestige bias model based on second-order cues. In this model, the prestige value of each agent is defined as the number of times the agent was previously selected as a demonstrator. Therefore, the main difference from a first-order cue model is that the prestige value of each agent can change over time. Therefore, the probability that each agent selects a demonstrator *k* = {1, 2, …, *N*} at time *t*, *q*_*k*,*t*_, is defined as follows:2
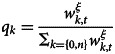


Here, *n* and *ξ* are the same as those in the first-order cues model; *w*_*k*,*t*_ is the prestige value of agent *k* at time *t*. In contrast to the first-order cues model, the prestige value of each agent is not a constant but a time-dependent variable. The prestige values of each agent at *t* = 0 were set to 1 for all agents. The demonstrators for each agent at *t* = 0 were determined randomly.

## 5. Simulation results

### 5.1. Prestige bias based on first-order cues

We present the simulation results of the prestige–bias model based on first-order cues. In the simulation, we set *T* = 1000 and the prestige bias *ξ* = [0.0, 1.0] in increments of 0.2. Population size is 100. The proportion of agents with a high prestige value in the population was set to 10%. The initial cultural traits and demonstrators for each agent were randomly provided.

**Figure 1.**
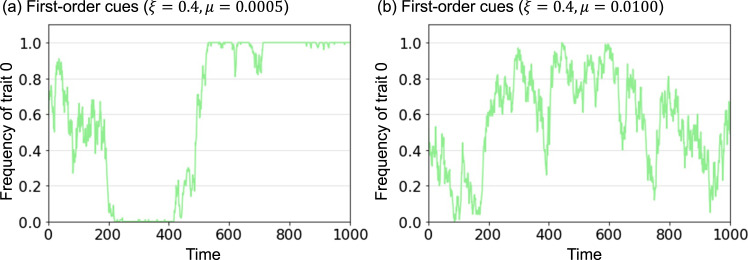
The typical dynamics of the frequency of cultural traits under prestige bias based on first-order cues (*ξ* = 0.4). (a) *μ* = 0.0005; (b) *μ* = 0.0100.

In this article, we present some example runs to describe the typical dynamics of cultural evolution, as we ran 100 simulations for each parameter. Figure 1 shows the dynamics of cultural evolution in the simulation with *ξ* = 0.4. When the mutation rate is relatively small (*μ* = 0.0005), the frequency of one of the two cultural traits rapidly converges to about 1.0. Yet, even when one trait became prevalent, it was sometimes replaced by the other. When the mutation rate is relatively high (*μ* = 0.0100), the dynamics continued to fluctuate and no clear prevalent trait was observed. The distribution of cultural traits in this model suggests that the model is susceptible to mutations.

### 5.2. Prestige bias based on second-order cues

Here, we present the simulation results of the prestige-bias model based on second-order cues. In the simulation, we set *T* = 1000 and the prestige bias *ξ* = [0.0, 1.0] in increments of 0.2. For all agents, the initial values of prestige were set to 1.0. Population size is 100. The initial cultural traits and demonstrators of each agent were randomly provided.

**Figure 2.**
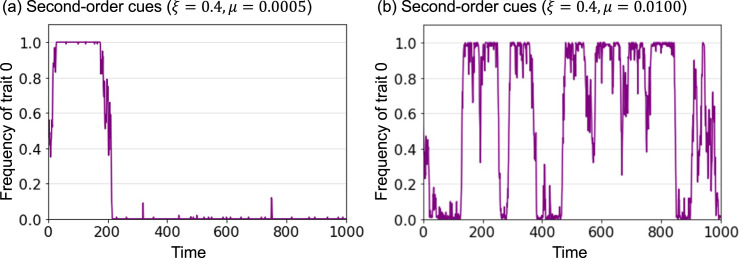
The typical dynamics of the frequency of cultural traits under prestige bias based on second-order cues (*ξ* = 0.4). (a and b) *μ* = 0.0005; (c and d) *μ* = 0.0100.

As in the previous section, we ran 100 simulations for each parameter; some examples are shown below. Figure 2 shows the dynamics of cultural evolution in the simulation with *ξ* = 0.4. As in the case of the first-order cues model, when the mutation rate is relatively low (*μ* = 0.0005), the frequency of one of the two cultural traits rapidly converges to approximately 1.0 and the prevalent trait changes over time. Compared with the first-order cues model, the prevalent traits seem to change abruptly; it takes only a short time for the turnover to occur (discussed in more detail later). Furthermore, even if the *μ* is relatively high prevalent traits are observed. Turnover occurs more frequently than in the case of a relatively low mutation rate.

### 5.3. Comparing two models

Our focus in this paper is to understand the cultural evolution shaped by prestige bias and how the two types of prestige bias differ in cultural dynamics. Accordingly, we discuss some details of the simulation results, focusing on a comparison between the two types of prestige bias.

#### 5.3.1. Comparison of turnover rate

As shown in the previous sections, both models show a turnover in prevalent cultural traits over time. We analysed the number of turnovers per run. For this analysis, we defined the prevalent state of each cultural trait as follows: we considered the prevalent state of cultural trait 0 as starting when the frequency of cultural trait 0 exceeded 0.9. The prevalent state was assumed to be maintained even when the frequency decreased below 0.9. The prevalent state of culture trait 1 begins when its frequency exceeds 0.9, that is, when the frequency of culture trait 0 falls below 0.1. We used 0.9, a value slightly lower than 1.0, as the criterion for the prevalent state to account for changes in traits owing to mutations. The number of turnovers was calculated by defining the prevalent state of a cultural trait in this manner. In each run, we removed the first 100 iterations as transient before calculation.

**Figure 3.**
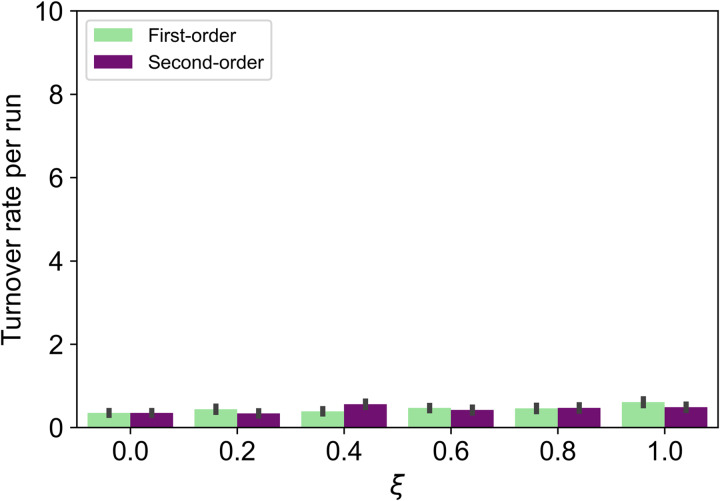
Turnover rate per run (*μ* = 0.0005). Error bars are standard errors.

**Figure 4.**
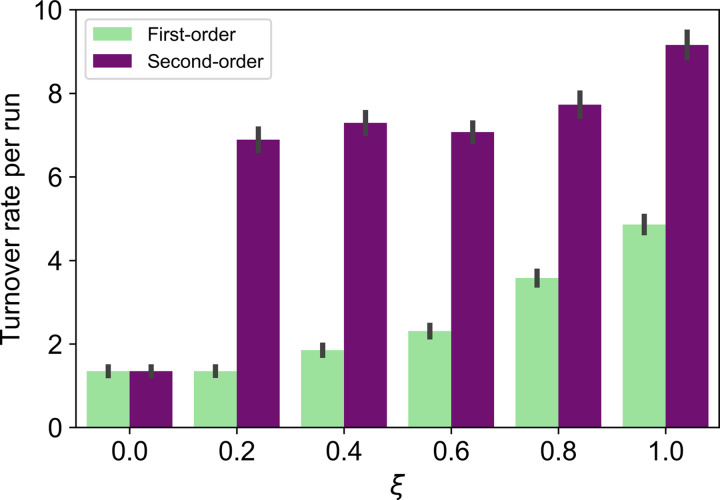
Turnover rate per run (*μ* = 0.0100). Error bars are standard errors.

We ran 100 simulations for each parameter setting and computed the mean number of turnovers per run and the standard errors. There was no clear difference in the turnover rate between the two models when the mutation rate was low (*μ* = 0.0005; Figure 3). However, when the mutation rate was high (*μ* = 0.0100), the two models showed a clear difference in the turnover rate (Figure 4). Figure 4 shows that, except when *ξ* is 0.0, the turnover rates in the second-order cues model are always higher than those in the first-order cues model. Similar results were obtained by changing the value of *μ* (see Supplementary Materials).

#### 5.3.2. Comparison of changes per time

Comparing the behaviour of the two models, changes in the frequency of cultural traits appeared to be more abrupt for the second-order cues model than for the first-order cues model (Figures 1 and 2). For quantitative analysis, we calculated the change in the frequency of trait 0 between time *t* and *t* + 1 (absolute value). In each run, we removed the first 100 times as transients and calculated the change in the remaining 900 times. A larger change indicated that the trait frequency changed more rapidly.

**Figure 5.**
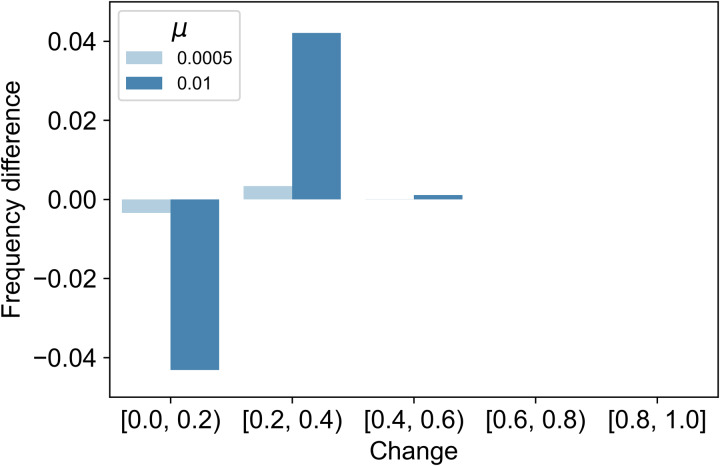
Differences of the change distribution between models (*ξ* = 0.4). *y*-Axis values greater than zero indicate greater frequency of observations in the second-order cues model.

**Figure 6.**
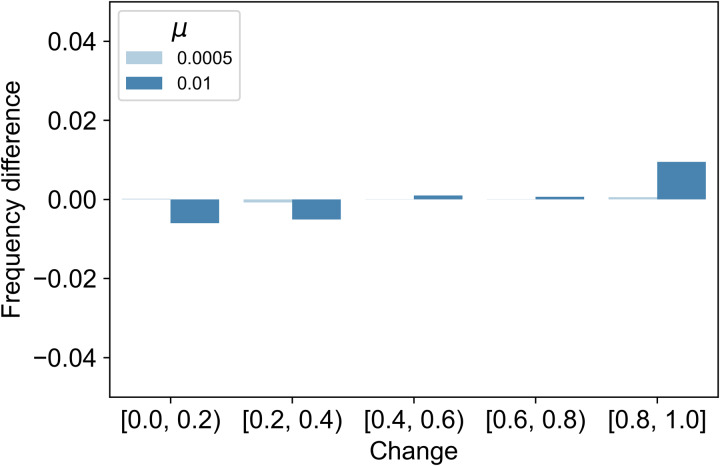
Differences of the change distribution between models (*ξ* = 1.0). *y*-Axis values greater than zero indicate greater frequency of observations in the second-order cues model.

The changes were classified in 0.2 increments and their distributions are shown in Tables S4–9. To examine the impact of *ξ* and *μ* values on the change per time, we analysed the change values of each. In addition, to examine the difference in the magnitude of change between models, we calculate the difference in the frequency of each change bin in the second-order cues model minus that in the first-order cues model (i.e. second-order column minus first-order column in Tables S4–9). Here we show the results for *ξ* = 0.4, *μ* = 0.0005, 0.0100 in Figure 5 and *ξ* = 1.0, *μ* = 0.0005, 0.0100 in Figure 6. When *ξ* = 0.4, changes above 0.2 are more likely to occur in the second-order cues model than in the first-order cues model (Figure 5). When *ξ* = 1.0, changes above 0.4 are more likely to occur in the second-order cues model than in the first-order cues model (Figure 6). In this case, even a change of about 0.8–1.0 per time can occur for second-order cues.

#### 5.3.3. Comparison of the prevalence formation

When the mutation rate is high, the prevalent cultural traits are formed in the second-order cues model but not in the first-order cues model (Figures 1 and 2). We drew a distribution of the frequency of cultural traits from all runs and times (the first 100 times were removed as transient). If the distribution has a salient peak around a frequency of 0.0 and/or 1.0, the prevalent traits appear.

**Figure 7.**
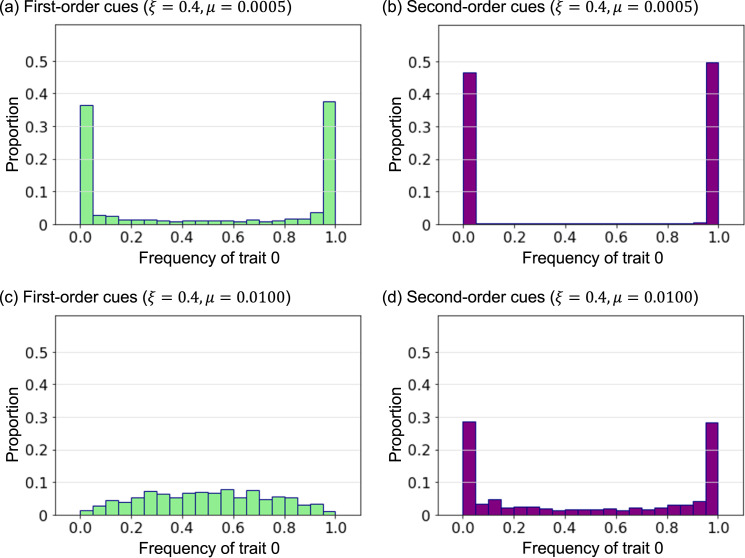
The distribution of frequency of the cultural trait. Each graph is given from 100 runs and 900 times. (a) First-order cues model with *ξ* = 0.4 and *μ* = 0.0005; (b) second-order cues model with *ξ* = 0.4 and *μ* = 0.0005; (c) first-order cues model with *ξ* = 0.4 and *μ* = 0.0100; and (d) second-order cues model with *ξ* = 0.4 and *μ* = 0.0100.

When the mutation rate is low, almost all runs exhibited prevalent cultural traits at almost all times (Figure 7a and b). When the mutation rate is high, the prevalent trait is no longer formed in the first-order cues model (Figure 7c); there were no salient peaks at 0.0 and 1.0 in the distribution. In contrast, the prevalent cultural trait is formed in the second-order cues model, even when the mutation rate is high (Figure 7d). Similar results were reproduced by changing the value of *ξ* and *μ* (see the Supplementary Materials).

## 6. General discussion

### 6.1. Difference between two models in cultural evolution

We reviewed the definitions of prestige bias and classified them into two types: first- and second-order cues model. First-order cues are attributed to the characteristics of demonstrators such as their intelligent appearances. Second-order cues are attributed to the behaviour of observers, such as how much attention they pay to demonstrators. To advance the theoretical understanding of prestige-biased social learning, we constructed two types of computational models based on different definitions and compared their cultural evolutions.

We investigated whether cultural traits were prevalent in the population and the frequency of turnover. We varied the mutation rate and observed differences between the models. In the simulations, prevalence and turnover were observed in many cases, except when the mutation rate was high in the first-order cues model. When the mutation rate was low, there was a minor difference in the prevalence and turnover rates between the two models. When the mutation rate was high in the second-order cues model, a trait became prevalent for some time but frequent turnover was observed. In contrast, when the mutation rate was high in the first-order cues model, the traits fluctuated and rarely became prevalent. When the mutation rate was high, the differences between the models were also pronounced in the frequency change of the cultural traits over time. In this case, the frequency changes in the second-order cues model were large, sometimes triggering a turnover even in one time (frequency change [0.8, 1.0]). These results suggest that turnover does not always result from the accumulation of small changes, but from rapid changes over short periods of time.

Cultural traits can be fixed in a very short time through social learning biases. Our results are not surprising, but we have shown that the two models of prestige bias based on different definitions can lead to different cultural evolutions. Different cultural patterns are due to different distributions of prestige. In the first-order cues model, 10 individuals (10% of the population) had prestige values 10 times higher than the remaining 90 individuals. All the prestigious individuals had the same prestige values. Therefore, a change in the cultural traits of a prestigious individual has a limited influence on the population. Therefore, a trait cannot be considered prevalent if its mutation rate is high. In contrast, in the second-order cues model, prestige values were not uniform and a small proportion of individuals dominated high prestige (see Supplementary Materials). A trait could be prevalent even if the mutation rate is high, because a mutation in the cultural trait of a prestigious individual has a large effect on the population. We are the first to propose a second-order cues model and demonstrate that the two definitions of prestige bias should be distinguished in terms of cultural evolution.

Our simulation highlights the importance of the distribution of prestige values in the cultural evolution. Prestige bias reduces the size of the demonstrator population (prestigious individuals) and makes the cultural evolution of neutral cultural traits more likely. This is a parallel phenomenon to neutral evolution through genetic drift. Our first-order cues model provided a smaller demonstrator population (10%), resulting in cultural evolution of neutral traits. The second-order cues model is likely to have a smaller demonstrator population than the first-order cues model, resulting in different cultural evolution. When *ξ* is 0.4 in the second-order cues model, the frequency of prestige values is often around 10 (see Figure S6). Given that the population size in the group was 100, it can be inferred that the number of demonstrators (individuals with high prestige) was often around 10. This is similar to the first-order cues model where 10 individuals have prestige values of 10. However, the non-flat distributions in the second-order cues model would probably have a similar effect as a smaller population of demonstrators. For example, assuming that there are 10 individuals with non-zero prestige in the second-order cues model, if there are individuals with prestige values of 4 and 14, the former is less likely to be chosen as a demonstrator. This leads to a similar condition where the effective number of demonstrators is less than 10. Consequently, the second-order cues model showed a greater temporal difference in trait frequency than the first-order cues model. This comparison suggests that the difference between the two models lies in the inequality of prestige values rather than in whether prestige values change over time. Essentially, allowing the agents’ prestige values to be sampled from a normal distribution can lead to a cultural evolution similar to that of the second-order cues model, even when prestige does not change over time. However, the validity of the assumption that prestige values vary requires further discussion. Although within an individual, it is unlikely that the perception of prestige will be the same for several others, given the inter-individual variance in the perception of prestige values for the same individual, it may be plausible to assume that the perception of prestige values for several others will be the same on average.

### 6.2. Application to empirical data

A promising direction for our study is the application of our model to empirical data. We discuss our research and its counterparts in the real world. The present study shows that high mutation rates and second-order cues models lead to very rapid cultural evolution and frequent turnover. Typically, mutations are simply treated as errors in models of cultural evolution. The present study suggests that simulations with high mutation rates simulate situations where behaviour is unimportant to the individual and can be easily changed. Rapid cultural change in second-order cues models is similar to trends on social networking sites. It would be caused by the presence of influencers with strong influence and behaviour that is not important to the individual. This would correspond to the rapid cultural evolution and turnover driven by non-uniform distribution of prestige and high mutation rates. In contrast, a relatively slow cultural evolution was observed in our first-order cue model. A flat distribution of prestige would be considered a real-life situation where no clear order between prestigious individuals is known. When a beginner buys a tennis racket, in the absence of other strong preferences, he is likely to choose a racket from the same manufacturer as that used by a famous player. Yet it is unclear whether he should choose the racket used by player A or player B, who is also famous (many small factors are involved in the final decision, but we consider them as random effects). Our first-order cues model could represent a situation where prestige information is used to narrow down a large number of candidates (10% of high prestige individuals) and one of them is chosen at random. In this case, the cultural evolution is slow, and if it is not an important behaviour (high mutation rate), then several traits will evolve in the population.

We define prestige bias based on previous studies and formulate a model of cultural evolution driven by prestige bias. By applying our models to empirical data, we can analyse the contribution of prestige bias to cultural evolution. Acerbi and Bentley (2014) focused on the turnover phenomenon in cultural evolution and analysed the contribution of biased social learning. Turnover in cultural evolution, a ubiquitous feature of contemporary culture, occurs when high-frequency traits are replaced in the ranked list of cultural traits over time; sometimes, new or previously rare traits are ranked high on the list. Our results show that both the first- and second-order cue models show turnover and dynamic replacement of the majority trait, even when the bias and mutation rates are low. Acerbi and Bentley (2014) focus on the contributions of content and context biases (conformity and anti-conformity biases) to cultural evolution. However, in some cases, such as the ‘Angelina effect’, prestige bias plays a crucial role in the transmission of cultural traits (Henrich, 2015). Especially in the case of the transmission of pop culture, fashion items and information dissemination on social networking service, the impact of celebrity (‘pop icon’ or ‘fashion icon’) or ‘influencer’ may be more powerful. Analysing the contribution of prestige bias allows us to address how cultural traits propagate in these phenomena.

To apply our models to the empirical data, we must refine them in several ways. First, the model focuses on limited conditions in which only two traits exist. The invention rate must be introduced to represent the emergence of novel traits over time. Furthermore, in our second-order cues model, prestige was concentrated on one individual; that is, almost all individuals chose the same individual as a demonstrator, and this did not change over time. This is impractical when models are applied to empirical data. In our second-order cues model, individuals choose a demonstrator based on the proportion of their prestige. In contrast, the selection of demonstrators can be improved by defining a ‘top list’ of high-prestige individuals and uniformly and randomly selecting individuals from the top list (Acerbi & Bentley, 2014). This would allow the model to represent a moderate diffusion of demonstrators while still representing the emergence of prestige and the concentration of imitation on high-prestige individuals in a manner consistent with reality.

### 6.3. Prestige bias and cultural evolution: Cumulative cultural evolution and maladaptive cultural evolution

Prestige-biased social learning leads to two important types of cultural evolution: cumulative cultural evolution, the gradual development of culture over generations, and maladaptive cultural evolution, the spread of culture that is not adaptive to the individual. If a positive correlation exists between prestige and individual success, prestige-biased social learning leads to the cumulative cultural evolution of beneficial traits (Henrich & Boyd, 2002). This is because prestige bias is considered identical to the success/payoff bias when the positive correlation between prestige and success is strong. Social learning biased towards success (payoff) leads to the cumulative cultural evolution of beneficial traits (Mesoudi, 2016). A positive correlation between prestige and success would lead to cumulative cultural evolution is no great mystery, but the question is how a positive correlation would arise. In first-order cues model, researchers give each individual's prestige in advance as an exogenous variable. To study the emergence of a correlation between prestige and success, the second-order cues model approach would be needed.

The prestige of the individual is considered to be rooted in the success achieved through his or her skills and knowledge (Henrich & Gil-White, 2001). However, in our society, there are some cases in which there is no correlation between prestige and success. For example, individuals called social media influencers whose actual abilities are unknown reportedly exert a significant influence. If there is no positive correlation between prestige and individual success, prestige-biased social learning may lead to maladaptive cultural evolution. For example, Ihara (2008) modelled a situation in which there was a negative correlation between prestige and reproductive success. In this study, prestige was modelled based on first-order cues and individuals earned prestige through prestige-seeking behaviour. Although prestige-seeking behaviour reduces reproductive success, it has evolved culturally because individuals are more likely to imitate the behaviour of individuals with high prestige. Boyd and Richerson (1988) argued that if high prestige is associated with non-adaptive and extreme cultural traits, runaway processes can explain non-adaptive cultural evolution. These studies show that maladaptive behaviours that are not common in biological evolution alone are common in cultural evolution. However, it does not explain why the correlation is lost first. Previous models established the relationship between prestige and success. To study the dynamics of the maintenance or loss of the correlation between prestige and success, it would be useful to have a model that can handle the dynamics of prestige emergence and change, such as our second-order cues model.

### 6.4. The evolution of prestige bias

Building formal prestige bias models allows for research in several promising directions. The first is to examine the conditions under which prestige bias evolves. Here, we discuss the theoretical hypotheses regarding evolutionary conditions and future directions for modelling. Prestige information is considered an indirect indicator when the demonstrator's payoff is unknown (Henrich, 2015). Henrich and Gil-White argue that the tendency to copy the behaviour of prestigious demonstrators evolves when both individual learning and direct assessment of the demonstrator's knowledge/skills are costly or difficult (Henrich & Gil-White, 2001; Jimenéz & Mesoudi, 2019). Laboratory experiments have shown that people use prestige bias under these conditions (Atkisson et al., 2012; Brand et al., 2020). Therefore, prestige bias may be adaptive in a given environment, even in the presence of other biases such as payoff bias. To test this hypothesis on the evolution of biases, we need to add several features to our model: the introduction of payoffs from cultural traits and the difficulty of evaluating information about payoffs (e.g. owing to variance in payoffs or the number of cultural traits). It would also be worthwhile to investigate the conditions under which prestige bias evolves, considering other factors: the degree of association between prestige and payoffs and the exploration–exploitation trade-off between non-social and social learning. This discussion on the evolution of prestige bias implicitly assumes a model in which there can be both bias against high payoff traits and prestige bias.

## Supporting information

Nakata et al. supplementary material Nakata et al. supplementary material

## Data Availability

See above.

## Supplementary material

To view supplementary material for this article, please visit https://doi.org/10.1017/ehs.2024.12

## Research transparency and reproducibility

All simulation codes are available at https://github.com/SeiyaNAKATA/Computational-modeling-of-prestige-biased-social-learning.git
