# Supplementary material for: Formalising prestige bias: Differences between models with first-order and second-order cues
Source: Evol Hum Sci. 2024 Mar 20;6:e21. doi: 10.1017/ehs.2024.12 (PMC11058518; doi:10.1017/ehs.2024.12)
Supplement: Nakata et al. supplementary material [file S2513843X24000124sup001.docx]

Supplementary files of “Computational modelling of prestige-biased social learning: Differences between models with first-order cues and second-order cues”

Seiya Nakata^1, *^, Akira Masumi^2^, and Genta Toya^3^

1 International Research Center for Neurointelligence (WPI-IRCN), The University of Tokyo, Tokyo, Japan

2 School of Knowledge Science, Japan Advanced Institute of Science and Technology, Ishikawa, Japan

3 Research Center for Advanced Science and Technology, The University of Tokyo, Tokyo, Japan

**Contents**

S1. Turnover rate (with different values of mutation rate)

S2. Distribution of changes in trait frequency per time (with different values of mutation rate and bias)

S3. The distribution of the frequency of the cultural trait (with different values of mutation rate and bias)

S4. The distribution of prestige values in the second-order cues model

# S1. Turnover rate (with different values of mutation rate)

In the main text, we reported the turnover rate per run for simulations with mutation rates of 0.0005 and 0.01. Here we present results for mutation rates of 0.0030, 0.0055, and 0.0080 (Fig. S1-3). The trends are similar to those reported in the main text. Except when $\xi$ is 0.0, the turnover rates in the second-order cues model are always higher than those in the first-order cues model. The differences in turnover rates became larger as the mutation rate increased.


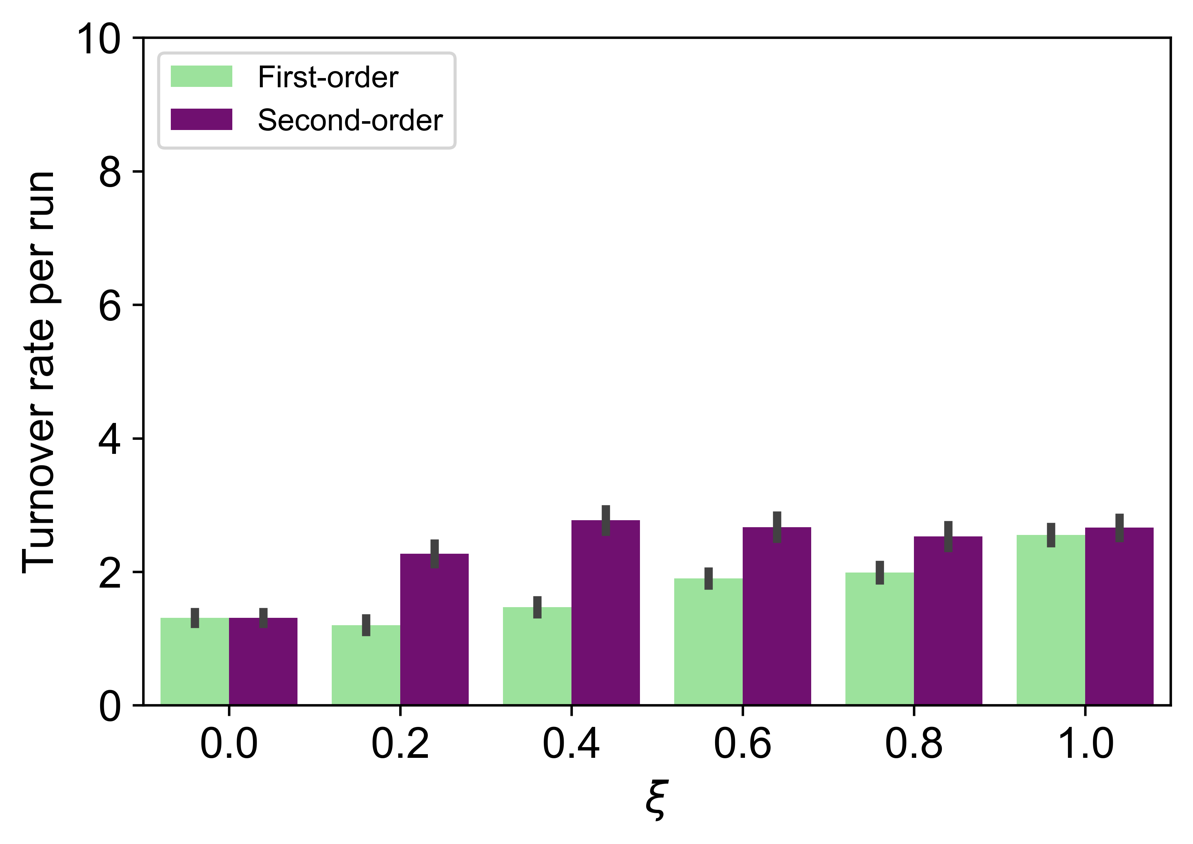


Fig. S1. Turnover rate per run ($\mu=0.0030$). Error bars represent standard error.


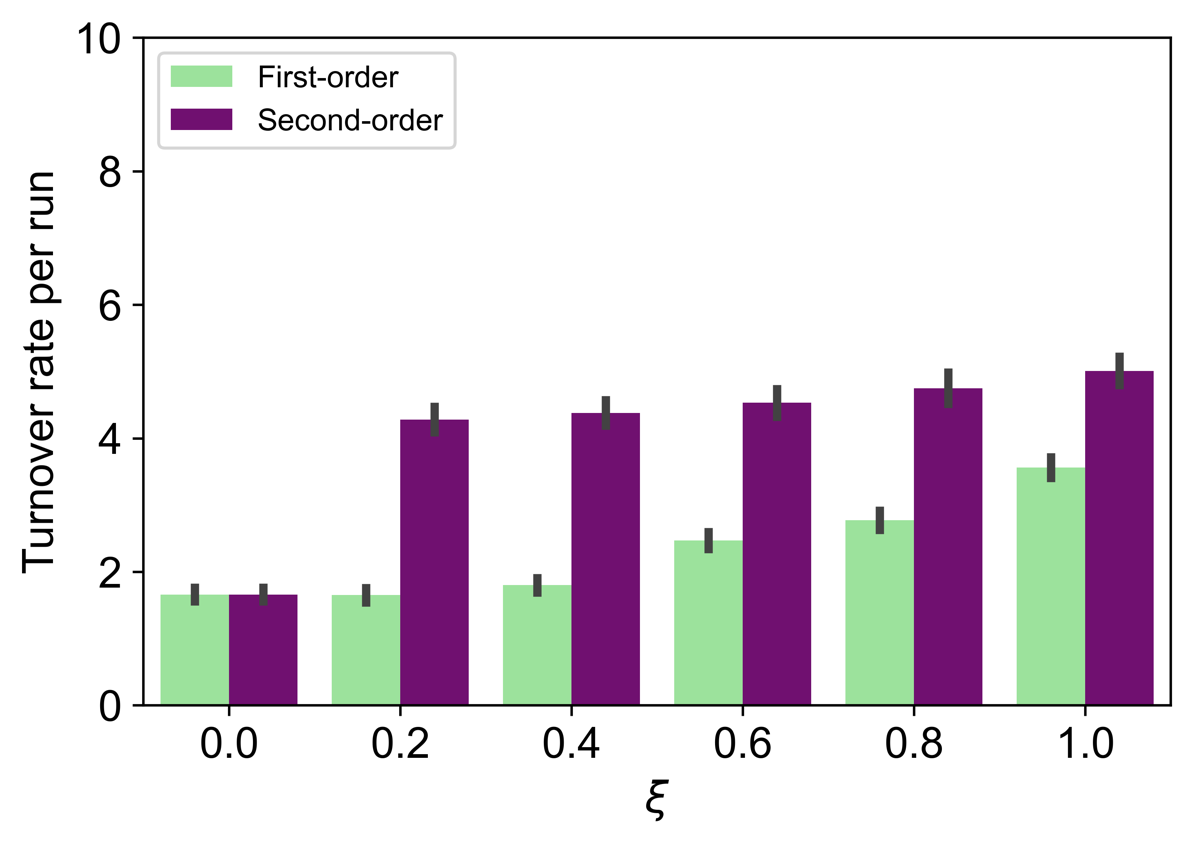


Fig. S2. Turnover rate per run ($\mu=0.0055$). Error bars represent standard error.


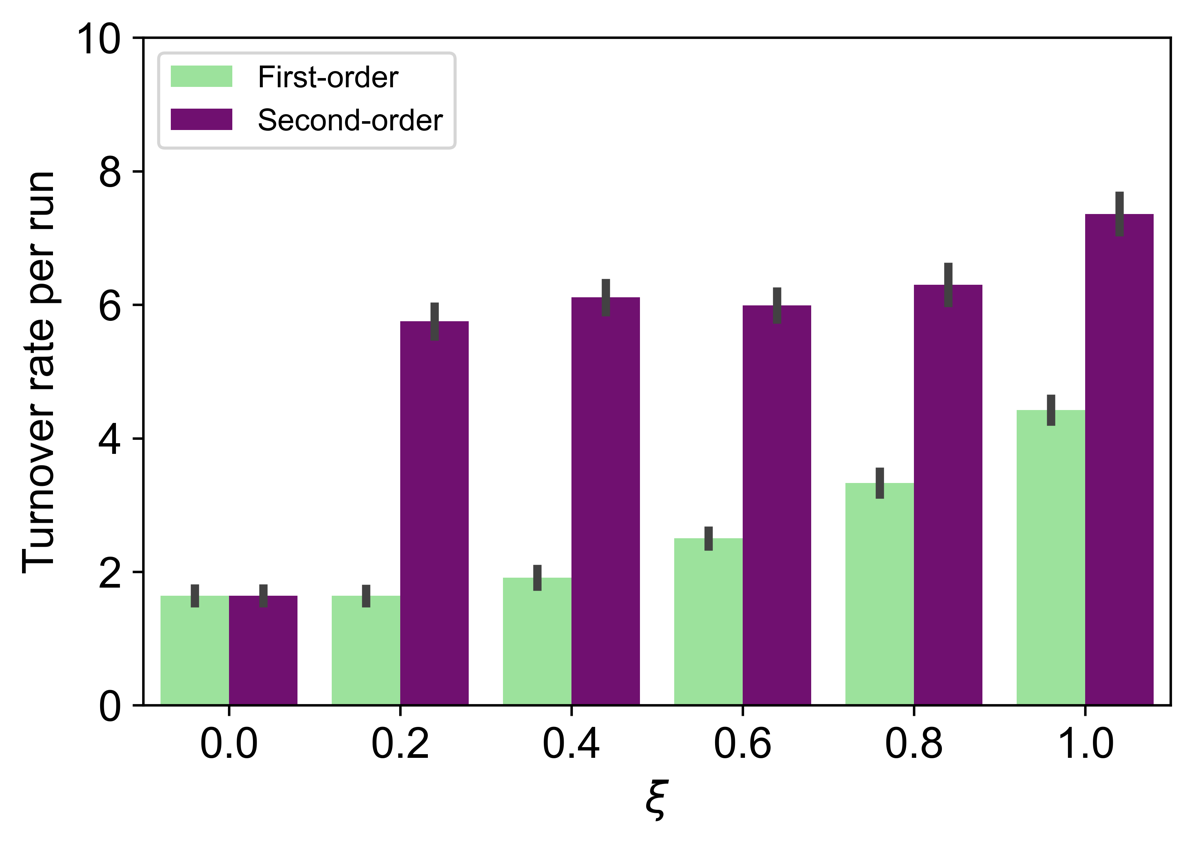


Fig. S3. Turnover rate per run ($\mu=0.008$ $0080$). Error bars represent standard error.


# S2. Distribution of changes in trait frequency per time (with different values of mutation rate and bias)

In the main text, we reported the changes in trait frequency per time for simulations with [$\xi=0.4, \mu=0.0005$], [$\xi=0.4, \mu=0.0100$], [$\xi=1.0, \mu=0.0005$], and [$\xi=1.0, \mu=0.01$00]. Here we show the results when $\xi$ (0.0, 0.2, 0.4, 0.6, 0.8, and 1.0) and $\mu$ (0.0005, 0.0030, 0.0055, 0.0080, and 0.0100) are changed to different values (Tables S4-9). Consistent with the results in the main text, the change per time in the frequency of the cultural trait tends to be larger for the second-order cues model than for the first-order cues model in each parameter regime.

Table S4. Distribution of changes in trait frequency per time ($\xi=0.0$)

| Mutation rate ($\mu$) | Change | First-order | Second-order |
| --- | --- | --- | --- |
| 0.0005 | [0.0, 0.2) | 1.00000 | 1.00000 |
|  | [0.2, 0.4) | 0.00000 | 0.00000 |
|  | [0.4, 0.6) | 0.00000 | 0.00000 |
|  | [0.6, 0.8) | 0.00000 | 0.00000 |
|  | [0.8, 1.0] | 0.00000 | 0.00000 |
| 0. 0030 | [0.0, 0.2) | 0.99999 | 0.99999 |
|  | [0.2, 0.4) | 0.00001 | 0.00001 |
|  | [0.4, 0.6) | 0.00000 | 0.00000 |
|  | [0.6, 0.8) | 0.00000 | 0.00000 |
|  | [0.8, 1.0] | 0.00000 | 0.00000 |
| 0.0055 | [0.0, 0.2) | 1.00000 | 1.00000 |
|  | [0.2, 0.4) | 0.00000 | 0.00000 |
|  | [0.4, 0.6) | 0.00000 | 0.00000 |
|  | [0.6, 0.8) | 0.00000 | 0.00000 |
|  | [0.8, 1.0] | 0.00000 | 0.00000 |
| 0.0080 | [0.0, 0.2) | 0.99994 | 0.99994 |
|  | [0.2, 0.4) | 0.00006 | 0.00006 |
|  | [0.4, 0.6) | 0.00000 | 0.00000 |
|  | [0.6, 0.8) | 0.00000 | 0.00000 |
|  | [0.8, 1.0] | 0.00000 | 0.00000 |
| 0.0100 | [0.0, 0.2) | 0.99994 | 0.99994 |
|  | [0.2, 0.4) | 0.00006 | 0.00006 |
|  | [0.4, 0.6) | 0.00000 | 0.00000 |
|  | [0.6, 0.8) | 0.00000 | 0.00000 |
|  | [0.8, 1.0] | 0.00000 | 0.00000 |

Table S5. Distribution of changes in trait frequency per time ($\xi=0.2$)

| Mutation rate ($\mu$) | Change | First-order | Second-order |
| --- | --- | --- | --- |
| 0.0005 | [0.0, 0.2) | 0.99999 | 0.99768 |
|  | [0.2, 0.4) | 0.00001 | 0.00231 |
|  | [0.4, 0.6) | 0.00000 | 0.00001 |
|  | [0.6, 0.8) | 0.00000 | 0.00000 |
|  | [0.8, 1.0] | 0.00000 | 0.00000 |
| 0. 0030 | [0.0, 0.2) | 0.99998 | 0.98630 |
|  | [0.2, 0.4) | 0.00002 | 0.01347 |
|  | [0.4, 0.6) | 0.00000 | 0.00023 |
|  | [0.6, 0.8) | 0.00000 | 0.00000 |
|  | [0.8, 1.0] | 0.00000 | 0.00000 |
| 0.0055 | [0.0, 0.2) | 0.99994 | 0.97793 |
|  | [0.2, 0.4) | 0.00006 | 0.02169 |
|  | [0.4, 0.6) | 0.00000 | 0.00038 |
|  | [0.6, 0.8) | 0.00000 | 0.00000 |
|  | [0.8, 1.0] | 0.00000 | 0.00000 |
| 0.0080 | [0.0, 0.2) | 0.99994 | 0.96996 |
|  | [0.2, 0.4) | 0.00006 | 0.02959 |
|  | [0.4, 0.6) | 0.00000 | 0.00046 |
|  | [0.6, 0.8) | 0.00000 | 0.00000 |
|  | [0.8, 1.0] | 0.00000 | 0.00000 |
| 0.0100 | [0.0, 0.2) | 0.99996 | 0.96391 |
|  | [0.2, 0.4) | 0.00004 | 0.0356 |
|  | [0.4, 0.6) | 0.00000 | 0.00049 |
|  | [0.6, 0.8) | 0.00000 | 0.00000 |
|  | [0.8, 1.0] | 0.00000 | 0.00000 |

Table S6. Distribution of changes in trait frequency per time ($\xi=0.4$)

| Mutation rate ($\mu$) | Change | First-order | Second-order |
| --- | --- | --- | --- |
| 0.0005 | [0.0, 0.2) | 0.99998 | 0.9969 |
|  | [0.2, 0.4) | 0.00002 | 0.00303 |
|  | [0.4, 0.6) | 0.00000 | 0.00007 |
|  | [0.6, 0.8) | 0.00000 | 0.00000 |
|  | [0.8, 1.0] | 0.00000 | 0.00000 |
| 0. 0030 | [0.0, 0.2) | 0.99999 | 0.98476 |
|  | [0.2, 0.4) | 0.00001 | 0.01479 |
|  | [0.4, 0.6) | 0.00000 | 0.00044 |
|  | [0.6, 0.8) | 0.00000 | 0.00001 |
|  | [0.8, 1.0] | 0.00000 | 0.00000 |
| 0.0055 | [0.0, 0.2) | 0.99996 | 0.97558 |
|  | [0.2, 0.4) | 0.00004 | 0.02376 |
|  | [0.4, 0.6) | 0.00000 | 0.00067 |
|  | [0.6, 0.8) | 0.00000 | 0.00000 |
|  | [0.8, 1.0] | 0.00000 | 0.00000 |
| 0.0080 | [0.0, 0.2) | 0.99997 | 0.96750 |
|  | [0.2, 0.4) | 0.00003 | 0.03158 |
|  | [0.4, 0.6) | 0.00000 | 0.00092 |
|  | [0.6, 0.8) | 0.00000 | 0.00000 |
|  | [0.8, 1.0] | 0.00000 | 0.00000 |
| 0.0100 | [0.0, 0.2) | 0.9999 | 0.96133 |
|  | [0.2, 0.4) | 0.0001 | 0.03768 |
|  | [0.4, 0.6) | 0.00000 | 0.00099 |
|  | [0.6, 0.8) | 0.00000 | 0.00000 |
|  | [0.8, 1.0] | 0.00000 | 0.00000 |

Table S7. Distribution of changes in trait frequency per time ($\xi=0.6$)

| Mutation rate ($\mu$) | Change | First-order | Second-order |
| --- | --- | --- | --- |
| 0.0005 | [0.0, 0.2) | 0.99997 | 0.99713 |
|  | [0.2, 0.4) | 0.00003 | 0.00278 |
|  | [0.4, 0.6) | 0.00000 | 0.00009 |
|  | [0.6, 0.8) | 0.00000 | 0.00000 |
|  | [0.8, 1.0] | 0.00000 | 0.00000 |
| 0. 0030 | [0.0, 0.2) | 0.99971 | 0.98311 |
|  | [0.2, 0.4) | 0.00029 | 0.01604 |
|  | [0.4, 0.6) | 0.00000 | 0.00084 |
|  | [0.6, 0.8) | 0.00000 | 0.00000 |
|  | [0.8, 1.0] | 0.00000 | 0.00000 |
| 0.0055 | [0.0, 0.2) | 0.99971 | 0.97088 |
|  | [0.2, 0.4) | 0.00029 | 0.02786 |
|  | [0.4, 0.6) | 0.00000 | 0.00124 |
|  | [0.6, 0.8) | 0.00000 | 0.00002 |
|  | [0.8, 1.0] | 0.00000 | 0.00000 |
| 0.0080 | [0.0, 0.2) | 0.99963 | 0.96164 |
|  | [0.2, 0.4) | 0.00037 | 0.03666 |
|  | [0.4, 0.6) | 0.00000 | 0.00168 |
|  | [0.6, 0.8) | 0.00000 | 0.00002 |
|  | [0.8, 1.0] | 0.00000 | 0.00000 |
| 0.0100 | [0.0, 0.2) | 0.99958 | 0.9554 |
|  | [0.2, 0.4) | 0.00042 | 0.04272 |
|  | [0.4, 0.6) | 0.00000 | 0.00184 |
|  | [0.6, 0.8) | 0.00000 | 0.00003 |
|  | [0.8, 1.0] | 0.00000 | 0.00000 |

Table S8. Distribution of changes in trait frequency per time ($\xi=0.8$)

| Mutation rate ($\mu$) | Change | First-order | Second-order |
| --- | --- | --- | --- |
| 0.0005 | [0.0, 0.2) | 0.99982 | 0.99582 |
|  | [0.2, 0.4) | 0.00018 | 0.00376 |
|  | [0.4, 0.6) | 0.00000 | 0.00036 |
|  | [0.6, 0.8) | 0.00000 | 0.00007 |
|  | [0.8, 1.0] | 0.00000 | 0.00000 |
| 0. 0030 | [0.0, 0.2) | 0.99908 | 0.97991 |
|  | [0.2, 0.4) | 0.00092 | 0.01824 |
|  | [0.4, 0.6) | 0.00000 | 0.00177 |
|  | [0.6, 0.8) | 0.00000 | 0.00008 |
|  | [0.8, 1.0] | 0.00000 | 0.00000 |
| 0.0055 | [0.0, 0.2) | 0.99841 | 0.96483 |
|  | [0.2, 0.4) | 0.00159 | 0.03170 |
|  | [0.4, 0.6) | 0.00000 | 0.00330 |
|  | [0.6, 0.8) | 0.00000 | 0.00017 |
|  | [0.8, 1.0] | 0.00000 | 0.00000 |
| 0.0080 | [0.0, 0.2) | 0.99831 | 0.95122 |
|  | [0.2, 0.4) | 0.00169 | 0.04416 |
|  | [0.4, 0.6) | 0.00000 | 0.00447 |
|  | [0.6, 0.8) | 0.00000 | 0.00016 |
|  | [0.8, 1.0] | 0.00000 | 0.00000 |
| 0.0100 | [0.0, 0.2) | 0.99834 | 0.94134 |
|  | [0.2, 0.4) | 0.00166 | 0.05310 |
|  | [0.4, 0.6) | 0.00000 | 0.00536 |
|  | [0.6, 0.8) | 0.00000 | 0.00019 |
|  | [0.8, 1.0] | 0.00000 | 0.00001 |

Table S9. Distribution of changes in trait frequency per time ($\xi=1.0$)

| Mutation rate ($\mu$) | Change | First-order | Second-order |
| --- | --- | --- | --- |
| 0.0005 | [0.0, 0.2) | 0.99930 | 0.99941 |
|  | [0.2, 0.4) | 0.00070 | 0.00003 |
|  | [0.4, 0.6) | 0.00000 | 0.00004 |
|  | [0.6, 0.8) | 0.00000 | 0.00002 |
|  | [0.8, 1.0] | 0.00000 | 0.00049 |
| 0. 0030 | [0.0, 0.2) | 0.99716 | 0.99696 |
|  | [0.2, 0.4) | 0.00283 | 0.00020 |
|  | [0.4, 0.6) | 0.00001 | 0.00013 |
|  | [0.6, 0.8) | 0.00000 | 0.00014 |
|  | [0.8, 1.0] | 0.00000 | 0.00257 |
| 0.0055 | [0.0, 0.2) | 0.99570 | 0.99414 |
|  | [0.2, 0.4) | 0.00430 | 0.00050 |
|  | [0.4, 0.6) | 0.00000 | 0.00030 |
|  | [0.6, 0.8) | 0.00000 | 0.00023 |
|  | [0.8, 1.0] | 0.00000 | 0.00482 |
| 0.0080 | [0.0, 0.2) | 0.99476 | 0.99150 |
|  | [0.2, 0.4) | 0.00524 | 0.00074 |
|  | [0.4, 0.6) | 0.00000 | 0.00043 |
|  | [0.6, 0.8) | 0.00000 | 0.00033 |
|  | [0.8, 1.0] | 0.00000 | 0.00699 |
| 0.0100 | [0.0, 0.2) | 0.99410 | 0.98923 |
|  | [0.2, 0.4) | 0.00590 | 0.00113 |
|  | [0.4, 0.6) | 0.00000 | 0.00066 |
|  | [0.6, 0.8) | 0.00000 | 0.00044 |
|  | [0.8, 1.0] | 0.00000 | 0.00853 |

# S3. The distribution of the frequency of the cultural trait (with different values of mutation rate and bias)

In the main text, we reported the frequency distribution of cultural traits from 100 runs and 900 times with the value of $\xi$ fixed at 0.04 and the value of $\mu$ fixed at 0.0005 and 0.0100. Here we show the results when $\xi$ (0.0, 0.2, 0.4, 0.6, 0.8, and 1.0) and $\mu$ (0.0005, 0.0030, 0.0055, 0.0080, and 0.0100) are changed to different values. Figure S4 shows the results of the first-order cues model and Figure S5 shows the results of the second-order cues model. Consistent with the results in the main text, when the mutation rate is high, the prevalent trait is no longer formed in the first-order cues model (Fig. S4); no salient peaks at 0.0 and 1.0 in the distribution. On the other hand, the prevalent cultural trait is formed in the second-order cues model even when the mutation rate is high (Fig. S5).


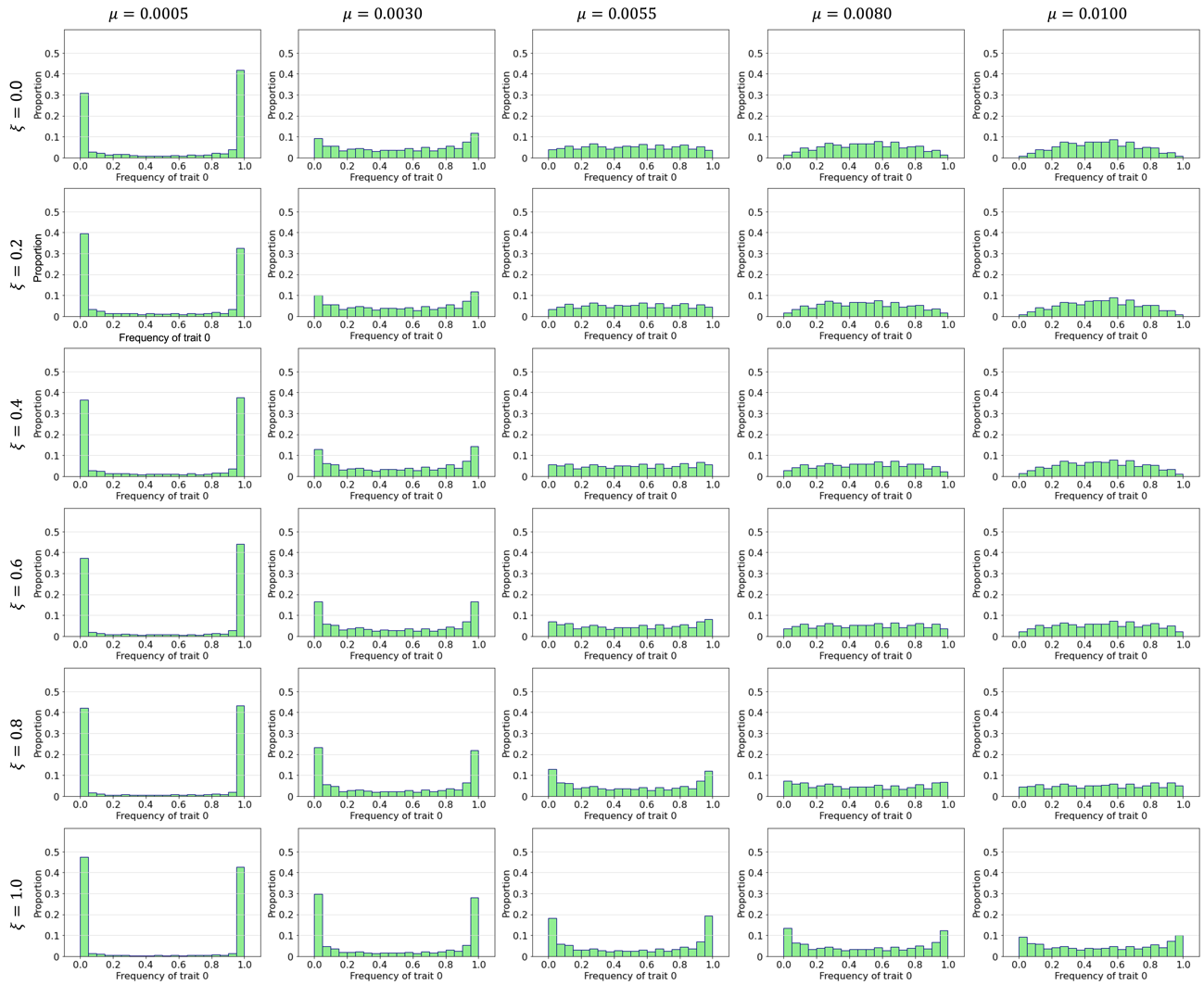


Fig S4. The distribution of the frequency of the cultural trait in the first-order cues model. The simulation results with different values of $\xi$ for each row and $\mu$ for each column. Each graph is given from 100 runs and 900 times.


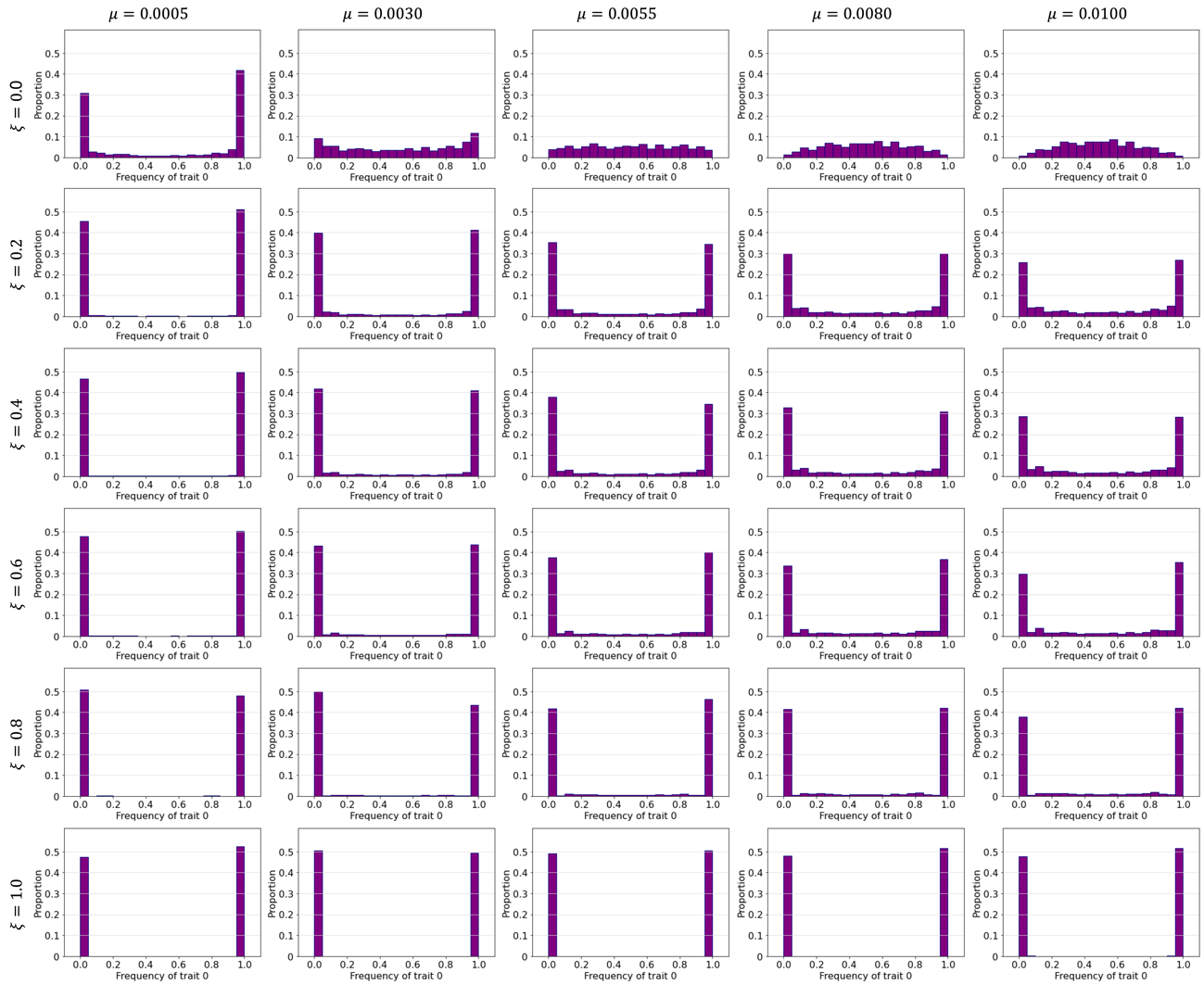


Fig S5. The distribution of the frequency of the cultural trait in the second-order cues model. The simulation results with different values of $\xi$ for each row and $\mu$ for each column. Each graph is given from 100 runs and 900 times.

# S4. The distribution of prestige values in the second-order cues model

In our second-order cues model, each agent's prestige value changes dynamically over time. Here we show the distribution of prestige values at time 1000 (the last time in our simulation) for each value of $\xi$ (The mutation rate values for cultural traits do not affect the distribution of prestige, and therefore do not need to be considered.). There are 100 agents per run in our simulation. To examine the average trend of the model, we pooled all the data for the 100 runs and calculated the frequency of the prestige values. In all simulations, a small fraction of the agents obtains high prestige values, while many other agents have the prestige values of 0. For the sake of clarity, in the histograms below, we have plotted the distribution without including data with zero prestige values.

We did not draw the distribution with $\xi$ =1.0 because in all runs only single agents monopolized prestige (i.e. prestige value 100). Each graph in Fig. S6 depicts a bell-shaped distribution, but the larger the value of $\xi$, the more the distribution moves to the right. Therefore, prestige was concentrated in a few agents and with increasing $\xi$.


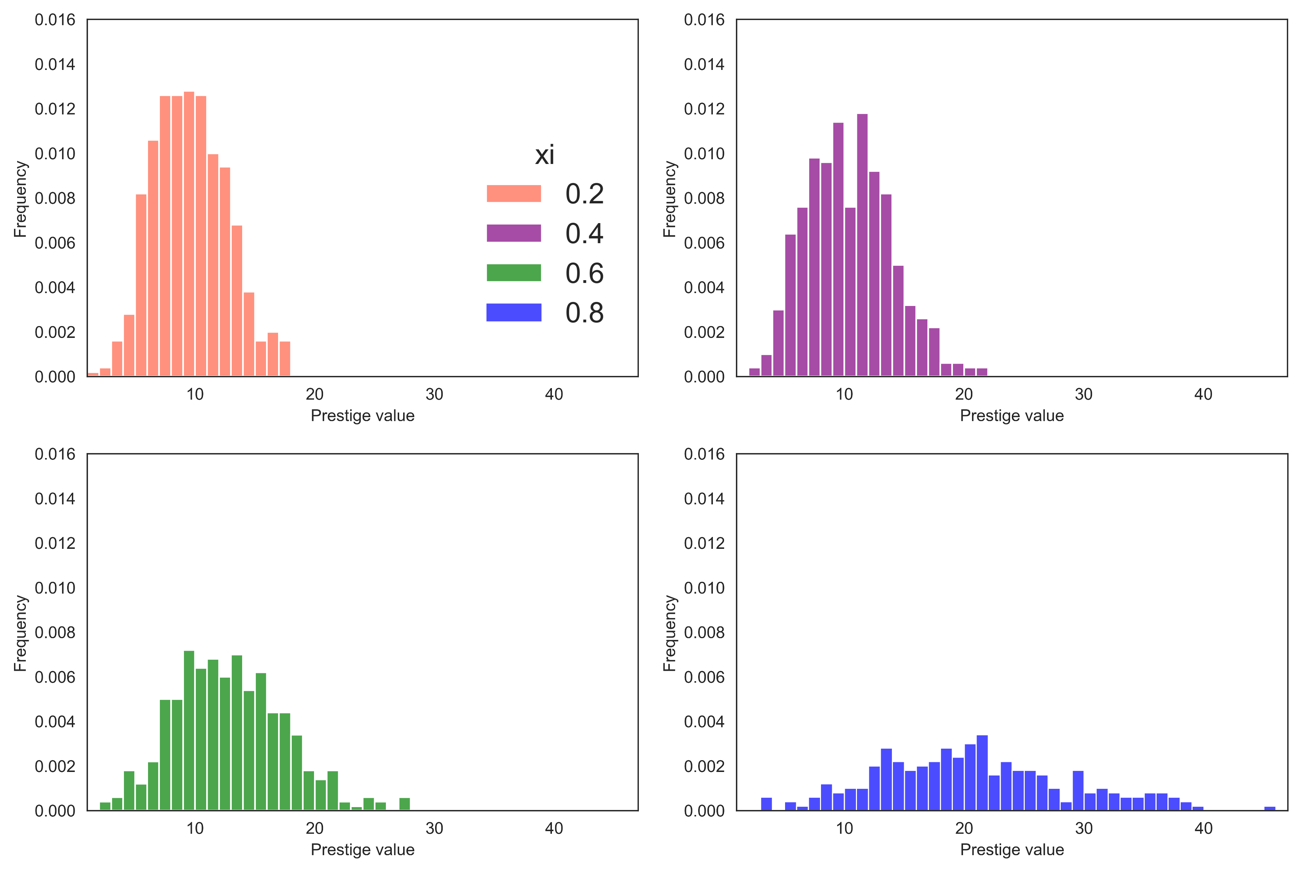


Fig. S6. The distribution of prestige values in the second-order cues model at time 1000. The upper left shows the $\xi$ = 0.2, the upper right 0.4, the lower left 0.6 and the lower right 0.8.
